# Social determinants of health associated with COVID-19 severity during pregnancy: a multinational cohort study (in the International Registry of Coronavirus Exposure in Pregnancy)

**DOI:** 10.1186/s12889-022-14532-8

**Published:** 2022-12-03

**Authors:** Jasmine A Mack, Erica A Voss, Rada Rusu, Meg Celine Hernandez, Sonia Hernandez-Diaz, Diego F Wyszynski, Shirley Sylvester, Rachael L DiSantostefano

**Affiliations:** 1 Janssen Research & Development, LLC, Titusville, NJ US; 2 Johnson & Johnson, Office of the Chief Medical Officer, New Brunswick, NJ US; 3 Harvard T.H. Chan School of Public Health, Boston, MA US; 4 Pregistry, LLC, London, England

**Keywords:** Pregnancy, COVID-19, Social determinants of health, Behaviors

## Abstract

**Background:**

The COVID-19 pandemic has caused morbidity and mortality, particularly among vulnerable populations. We aimed to assess social and demographic characteristics associated with COVID-19 severity among symptomatic participants during pregnancy.

**Methods:**

The International Registry of Coronavirus Exposure in Pregnancy is a multinational, longitudinal observational cohort study of adult participants tested for SARS-CoV-2 or who received clinical diagnosis of COVID-19 during pregnancy (NCT04366986). Disease severity status of mild, moderate, or severe was determined based on symptoms and healthcare utilization. Stratified by current versus recent pregnancy at enrollment, univariate mixed-effects logistic regression modeling was used to characterize association between social and demographic characteristics with COVID-19 severity, using a cumulative mixed effect model with country as a random effect.

**Results:**

The odds of developing more severe COVID-19 (odds ratio [95% confidence interval]) were higher among participants with lower socioeconomic status (poor: 2.72 [2.01,3.69]; lower-middle class: 2.07 [1.62,2.65] vs wealthy), among participants with lower educational attainment (high school: 1.68 [1.39,2.03]; < high school (1.77 [1.25,2.51] vs graduate education). Participants over 25 years of age had lower odds of severe COVID-19 versus participants < 25 years (25–34: 0.69 [0.56,0.85]; 35–50: 0.62 [0.48,0.80]). Employment in food services was also associated with increased odds of more severe COVID-19, whereas employment in healthcare and within home, and primiparity were associated with lower severity.

**Conclusions:**

Findings suggest that employment setting and economic status have strong associations with COVID-19 severity, which warrants considering social determinants of health in the context of assessing risk factors of more severe COVID-19 during pregnancy.

**Trial registration:**

IRCEP was registered with the European Network of Centres for Pharmacoepidemiology and Pharmacovigilance (ENCePP) [EUPAS37360] and clinicaltrials.gov [NCT04366986].

**Supplementary Information:**

The online version contains supplementary material available at 10.1186/s12889-022-14532-8.

## Introduction

The Coronavirus Disease 2019 (COVID-19) pandemic has exacerbated existing sociodemographic disparities during pregnancy. Previous studies have suggested that compared to non-pregnant individuals, pregnant individuals are at an increased risk of experiencing infection, severe illness, and severe maternal morbidity and mortality due to COVID-19 [1–3], which parallels pregnancy complications related to infection by other coronaviruses, such as Severe Acute Respiratory Syndrome (SARS) and Middle Eastern Respiratory Syndrome (MERS) [4]. Reported clinical factors related to infection and increased COVID-19 severity during pregnancy include pre-existing lung disease, obesity, cardiovascular disease, and diabetes [5, 6].

While some clinical risk factors have been associated with COVID-19 severity during pregnancy, environments where people work, live, and participate in leisure or social activities have direct impacts on health, especially in disease transmission and severity [7]. Ever since the launch of World Health Organization’s Commission on Social Determinants of Health (SDHs), more attention has been directed to the relationship between health and the societal context [8, 9]. Structural factors related to SDHs may play a role in the number of pregnant and recently pregnant people who experience severe COVID-19 disease globally. Furthermore, evidence demonstrates that SDHs can trigger inflammation in those that experience social inequity [10]. Thus, it is imperative to holistically evaluate and understand factors that affect health.

Previous studies have demonstrated that neighborhood level characteristics such as lower educational attainment and median household income were associated with COVID-19 illness severity during pregnancy [6]. Compared with pregnant participants who tested negative, participants who tested positive were younger and more likely to have public insurance, suggesting either a higher risk of infection in these groups or more frequent testing of asymptomatic individuals in older, privately insured groups [6, 11]. Having more children could be considered a proxy for overcrowding in a household and may culminate in difficulties social distancing, causing an increased risk of SARS-CoV-2 infection [6, 11]. Previous studies in 2020–2021 have focused on regional or within-country populations; therefore, global inequity and SDHs across countries have been less studied in terms of their impact on pregnant populations affected during the COVID-19 pandemic [12–14]. In this study across 41 countries, we aimed to assess social and demographic characteristics that are associated with COVID-19 severity during pregnancy in one of the largest COVID-19 pregnancy registries to date.

## Methods

### Study design and participants

Pregnant and recently pregnant participants enrolled in the International Registry of Coronavirus Exposure in Pregnancy (IRCEP, ircep.pregistry.com) by completing several online survey modules. IRCEP is a multinational, observational cohort study of adult participants who were tested for SARS-CoV-2, regardless of results, or had clinical diagnosis of COVID-19 during pregnancy. Questions concerning pre-pregnancy sociodemographic factors, maternal clinical characteristics, health outcomes, pregnancy/birth outcomes, and infant characteristics were asked. IRCEP was registered with the European Network of Centres for Pharmacoepidemiology and Pharmacovigilance (ENCePP) [EUPAS37360] and clinicaltrials.gov [NCT04366986], with additional design and cohort information published previously [15]. The Institutional Review Board of the Harvard T.H. Chan School of Public Health approved this study (IRB20-0622).

Participants with symptomatic COVID-19 any time during pregnancy who completed the demographics panel were included in our analysis (enrollment: June 24, 2020-March 18, 2021).

### Patient and public involvement

A focus group of pregnant and recently pregnant women in the United States participated in the review of the study surveys prior to the study launch. They evaluated the questions, the flow of the surveys, and the overall design of the study and provided important feedback that was subsequently used to improve both the content and the format of the web-based data collection tool. In addition, during the conduct of the study, participants were asked to provide comments about each survey module and their responses were carefully considered by the study team for possible use in the study.

### SARS-CoV-2 infection and COVID-19 severity outcomes

Participants self-reported COVID-19 symptoms, clinical diagnoses, and SARS-CoV-2 laboratory test results and were asked to upload de-identified medical records. For primary outcome, a formal COVID-19 test was not required, and a small proportion of the cases (6%) did not have test results available. This was more common at the beginning of the pandemic in some populations and/or countries; therefore, sensitivity analysis was utilized to assess whether availability of a positive test affected the results.

COVID-19 severity: mild, moderate, and severe was the primary outcome. Severity levels were determined based on symptoms and healthcare utilization using an algorithm similar to definitions provided by the Centers for Disease Control and Prevention (CDC) without clinical assessments (Additional file 1: Appendix 1). Severe COVID-19 was defined as admission to an intensive care unit (ICU); use of respiratory assistance; or being hospitalized, and experiencing organ failure, acute respiratory distress syndrome (ARDS), bluish lips/face, difficulty breathing, chest pain, or pneumonia. Participants with moderate COVID-19 had at least one of the following: abnormal chest X-ray/CT scan; ARDS, difficulty breathing, pneumonia, or visited a healthcare facility and experiencing either chest pain or bluish lips/face. Participants with moderate COVID-19 could have also visited a healthcare facility and experienced at least two of the following: high fever (≥ 38.0 °C or ≥ 100.4°F); sore throat/cough/runny nose; fatigue; headache; myalgia; loss of smell/taste; or gastrointestinal symptoms (i.e., abdominal pain, diarrhea, nausea, or vomiting). Lastly, mild COVID-19 was defined as experiencing at least one symptom and not meeting the definition of moderate or severe COVID-19.

### Social determinants of health

Social, demographic, and economic conditions that might influence health status included in our analysis were household size, employment at first COVID-19 symptoms, economic status, highest educational level, and World Bank income classification based on the participant’s country of residence [16]. Healthcare access was assessed by health insurance status and if participant had at least one healthcare visit during their current or recent pregnancy.

### COVID-19 preventive behaviors

Participants reported whether they elected to follow 17 different behaviors that could potentially lower the risk of COVID-19, including wearing a mask, avoiding crowded places, working from home, and handwashing, two weeks prior to diagnosis. These were included in our analysis as some were expected to be related to social and/or employment needs [17, 18].

### Health factors associated with COVID-19 severity

For completeness, pre-pregnancy self-reported health, pre-pregnancy body mass index (BMI), and medical comorbidities such as asthma, high blood pressure, and any other cardiovascular condition (e.g., heart failure, ventricular septal defects, arrhythmias) were included. Information about pregnancy and pregnancy history were also included, as they could potentially affect COVID-19 severity, including, whether pregnancy was considered high-risk, whether it was first pregnancy, and the trimester of COVID-19 exposure (reported at enrollment).

### Statistical analyses

Continuous variables are described as means and standard deviations (SD) and categorical variables are reported as frequencies and percentages. Stratified analyses were conducted by enrollment timing due to potential differences in COVID-19 severity between groups (pregnant vs. recently pregnant). Recently pregnant was defined as enrolling within 180 days following pregnancy. Prior to analysis, a chi-square test was conducted to examine whether pregnancy status at enrollment (pregnant vs. recently pregnant) was associated with COVID-19 severity. Participants in the recently pregnant group reported a higher frequency of severe COVID-19.

To determine the crude association between social and demographic characteristics and COVID-19 severity, univariate mixed-effects ordinal logistic regression models in the form of a cumulative link mixed effect model were performed. A random effect for country was added to the model to account for global differences during the pandemic. Odds ratios (OR), Wald-type 95% confidence intervals (95% CI), and statistical significance were reported for all parameters. To adjust for multiple testing given the number of social and demographic characteristics examined, Benjamini–Hochberg corrections were used to produce adjusted p-values. Ordinal logistic regression models assumed proportional odds, where odds ratios across all COVID-19 severity categories were equivalent (e.g., odds of having moderate vs mild COVID-19 was the same as the odds of having severe vs. moderate COVID-19). Model diagnostics were performed to test against the assumptions, including underlying proportional odds, or parallel assumptions, that allows for one set of coefficients for the COVID-19 severity levels. The proportional odds model assumption was evaluated using the Brant test [19]. All data analyses were performed using R [20].

## Results

**Fig. 1:**
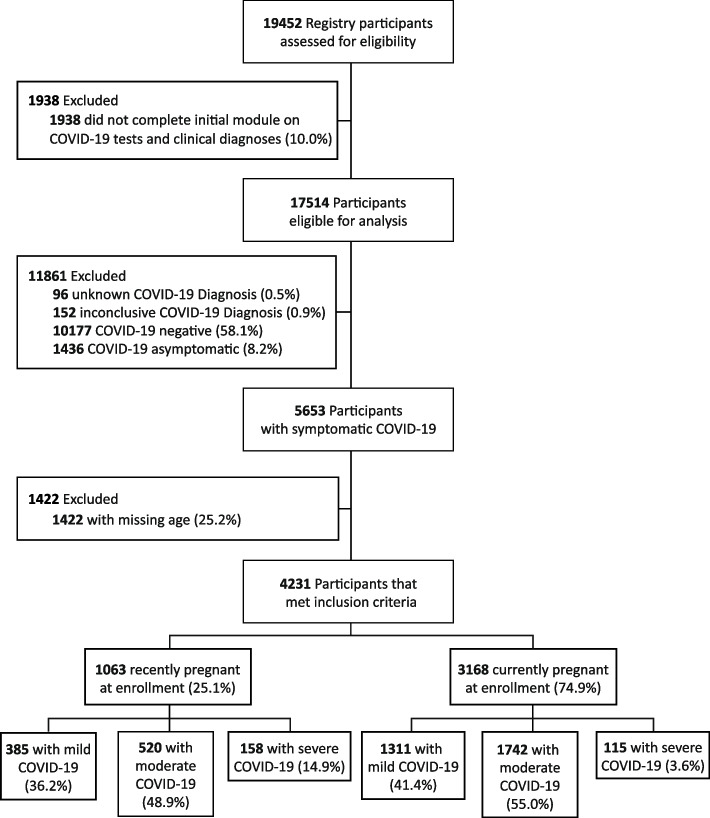
Study Inclusion Criteria Flowchart

Participants assessed for eligibility in this analysis were enrolled from June 24, 2020 to March 18, 2021. Of 19,452 participants (Fig. 1), 17,514 completed the initial SARS-CoV-2 status/testing module and were evaluated for inclusion into the analysis, with 11,861 of the samples being excluded for testing negative (*n* = 10,177), testing positive without any symptoms (*n* = 1,436) or having inconclusive or unknown SARS-CoV-2 status (*n* = 248). In all 5,653 participants who were considered positive for SARS-CoV-2 via test or clinical assessment and who exhibited symptoms, 4,231 completed the demographics panel. The 4,231 composed the analytical sample used to generate the results, with the primary analysis focusing on participants who were pregnant at enrollment (*n* = 3,168). Approximately, 25% of participants in the analytical sample enrolled after pregnancy (i.e., were in the recently pregnant group) (*n* = 1,063). The majority reported mild/moderate COVID-19 severity (94%, *n* = 4,231).

**Fig. 2:**
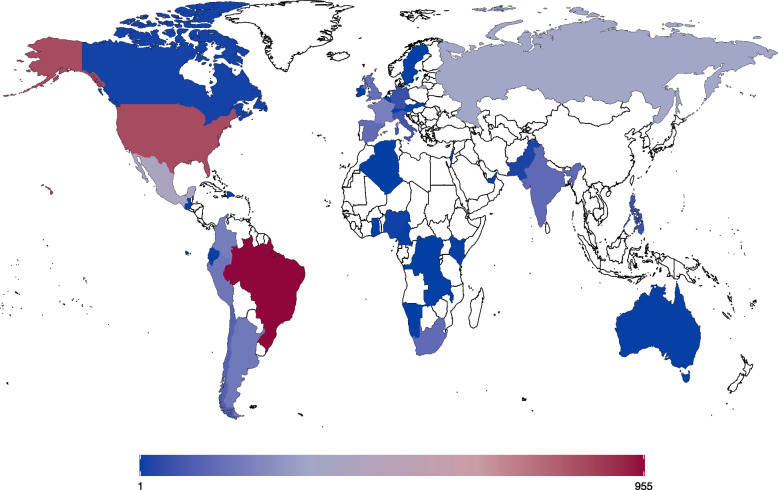
Number of Participants by Country (*n* = 4,231)

The analysis sample represented 41 countries (Fig. 2), with about half the sample representing Latin America and the Caribbean (48%), one-quarter representing Europe (23%), one-fifth representing North America (20%), and the remaining 10% representing the Middle East & Africa (4%), South Asia (4%), and East Asia & Pacific (2%) (based on the World Bank classification) [16]. The analysis cohort was diverse with respect to demographic, pre-pregnancy health, and reproductive history characteristics (Table 1). The majority were between 25–34 years of age (67%), and 46% reported being middle-class. At the time of the pandemic, about one-third of the analysis sample were unemployed (32%), one-fourth were working mostly from home (27%), and one-fifth were working in healthcare settings (19%). The majority reported excellent or very good health prior to pregnancy (66%).

**Table 1:** Characteristics of Participants

	Total (*n* = 4,231)	Currently Pregnant (*n* = 3,168)	Recently Pregnant (*n* = 1,063)
		*n* (%)	
Age at enrollment			
18–24	593 (14.0)	460 (14.5)	133 (12.5)
25–34	2,815 (66.5)	2,115 (66.8)	700 (65.9)
35–50	823 (19.5)	593 (18.7)	230 (21.6)
Employment at first COVID-19 symptoms			
None	1,341 (31.7)	897 (28.3)	444 (41.8)
Mostly from home	1,143 (27.0)	864 (27.3)	279 (26.2)
In healthcare	782 (18.5)	631 (19.9)	151 (14.2)
In an office	420 (9.9)	329 (10.4)	91 (8.6)
Other	407 (9.6)	337 (10.6)	70 (6.6)
In food services	95 (2.2)	80 (2.5)	15 (1.4)
Economic class			
Wealthy	535 (12.6)	396 (12.5)	139 (13.1)
Middle class	1,953 (46.2)	1,484 (46.8)	469 (44.1)
Lower-middle class	1,196 (28.3)	892 (28.2)	304 (28.6)
Poor	458 (10.8)	336 (10.6)	122 (11.5)
Highest educational level			
Graduate education	1,180 (27.9)	935 (29.5)	245 (23.0)
College	1,431 (33.8)	1,046 (33.0)	385 (36.2)
High school	1,343 (31.7)	994 (31.4)	349 (32.8)
Less than high school	236 (5.6)	164 (5.2)	72 (6.8)
Health coverage	3,558 (84.1)	2,668 (84.2)	890 (83.7)
Pre-pregnancy BMI			
< 25.0	1,596 (37.7)	1,216 (38.4)	380 (35.7)
25.0–29.9	911 (21.5)	670 (21.1)	241 (22.7)
≥ 30	821 (19.4)	603 (19.0)	218 (20.5)
Primiparous	1,571 (37.1)	1,178 (37.2)	393 (37.0)
High-risk pregnancy	959 (22.7)	685 (21.6)	274 (25.8)
Healthcare visit during pregnancy	3,580 (84.6)	2,662 (84.0)	918 (86.4)
Pre-pregnancy general health			
Excellent	1,563 (36.9)	1,142 (36.0)	421 (39.6)
Very good	1,236 (29.2)	935 (29.5)	301 (28.3)
Good	866 (20.5)	657 (20.7)	209 (19.7)
Fair/Poor	138 (3.3)	113 (3.6)	25 (2.4)
# Household (mean, SD)	2.51 (1.7)	2.45 (1.7)	2.70 (1.8)
Any household member positive	1,566 (37.0)	1,190 (37.6)	376 (35.4)

Note: Percentages are based on the number of participants in each column and may not add to 100% due to missing data or the ability to select more than one response

Data are *n* (%) unless stated otherwise. *BMI* Body mass index, *SD* Standard deviation

COVID-19 attributes and important comorbidities of participants are shown in (Table 2). There was a greater proportion of severe COVID-19 among those in the recently pregnant group than those in the pregnant group (15% vs. 4%). The most common symptoms reported were those related to upper respiratory infection: cough, sore throat, runny nose (82%), fatigue (77%), loss of smell and/or taste (75%), and myalgia (54%). Symptoms reported with < 50% frequency to characterize COVID-19 severity are reported in Additional file 1: Appendix 2. A small proportion of participants required hospitalization (7%), needed respiratory assistance (mechanical ventilation or extracorporeal membrane oxygenation [ECMO]; 2%), or were admitted to the ICU (2%) (Additional file 1: Appendix 2). Most participants reported some preventive behaviors in the two weeks prior to enrollment, including washing hands, wearing masks, and avoiding or postponing appointments or public gatherings. Exposure to COVID-19 occurred across the three trimesters but varied by pregnancy status at enrollment. Those with COVID-19 in the first trimester tended to enroll during pregnancy in the second or subsequent trimester while those with COVID-19 in the third trimester were more likely to enroll after the end of pregnancy (i.e., in the recently pregnant group).

**Table 2:** COVID-19 Attributes and Important Comorbidities of Participants

	Total (*n* = 4,231)	Currently Pregnant (*n* = 3,168)	Recently Pregnant (*n* = 1,063)
		*n* (%)	
COVID-19 Severity			
Mild	1,696 (40.1)	1,311 (41.4)	385 (36.2)
Moderate	2,262 (53.5)	1,742 (55.0)	520 (48.9)
Severe	273 (6.5)	115 (3.6)	158 (14.9)
COVID-19 Symptoms (> 50% frequency)			
Fatigue	3,244 (76.7)	2,451 (77.4)	793 (74.6)
Myalgia (muscle aches)	2,287 (54.1)	1,702 (53.7)	585 (55.0)
Loss of smell and/or taste	3,173 (75.0)	2,379 (75.1)	794 (74.7)
Runny Nose	2,270 (53.7)	1,776 (56.1)	494 (46.5)
Upper respiratory infection: cough, sore throat, runny nose	3,454 (81.6)	2,613 (82.5)	841 (79.1)
Diagnosed by health care professional with COVID-19	2,905 (68.7)	2,211 (69.8)	694 (65.3)
COVID-19 Preventative Behaviors (> 50% frequent)			
Avoided public spaces, gatherings, or crowds	2,863 (67.7)	2,126 (67.1)	737 (69.3)
Avoided contact with people who could be high-risk	2,583 (61.0)	1,939 (61.2)	644 (60.6)
Avoided eating at restaurants	2,273 (53.7)	1,662 (52.5)	611 (57.5)
Disinfected surfaces around me	2,498 (59.0)	1,845 (58.2)	653 (61.4)
Washed my hands with soap or used hand sanitizer several times per day	3,765 (89.0)	2,847 (89.9)	918 (86.4)
Wore a face mask	3,603 (85.2)	2,838 (89.6)	765 (72.0)
Trimester of COVID-19 exposure			
First	1,105 (26.1)	1,051 (33.2)	54 (5.1)
Second	1,550 (36.6)	1,325 (41.8)	225 (21.2)
Third	1,391 (32.9)	785 (24.8)	606 (57.0)
Medical Comorbidity			
Asthma	281 (6.6)	220 (6.9)	61 (5.7)
High blood pressure (chronic, pre-gestational)	140 (3.3)	98 (3.1)	42 (4.0)
Any cardiovascular condition (Heart failure, ventricular septal defects, arrhythmias)	82 (1.9)	61 (1.9)	21 (2.0)

Note: Percentages are based on the number of participants in each column and may not add to 100% due to missing data or ability to select more than one response

### Social and economic characteristics associated with COVID-19 severity

#### Participants pregnant at enrollment

**Fig. 3:**
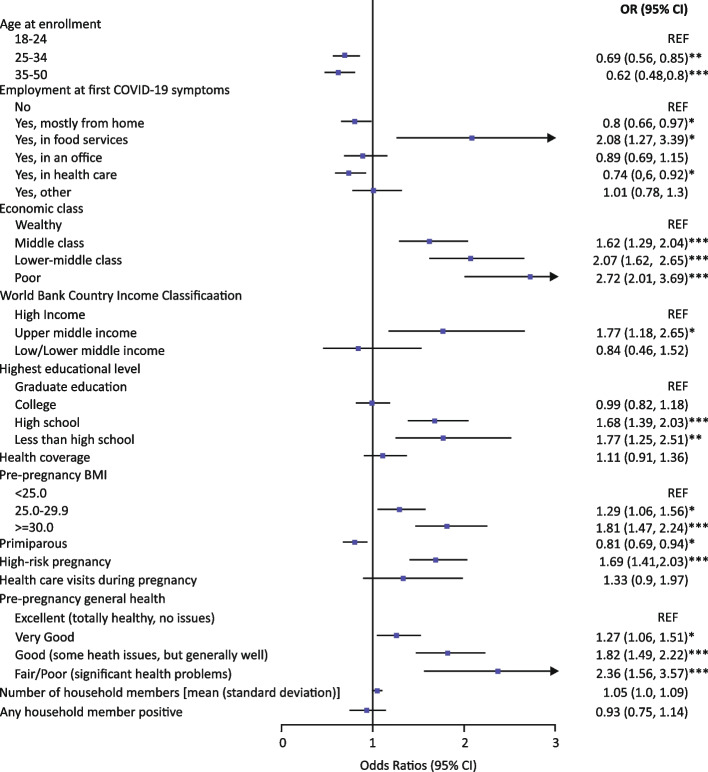
Currently Pregnant Participant Characteristics Associated with COVID-19 Severity. *p <  = 0.05, **p <  = 0.01. and ***p <  = 0.001. Note: Univariate mixed-effects ordinal logistic regression models with country as a random effect. Odds ratios (OR), Wald-type 95% confidence intervals, and Benjamini–Hochberg adjusted *p*-values were reported.

Among participants who were still pregnant at enrollment, some demographic and economic characteristics were associated with COVID-19 severity (Fig. 3). Participants with increased odds of more severe COVID-19 (i.e., mild to moderate, moderate to severe) were younger (below 25), where participants who were 25–34 years old and 35–50 years old had 31% (OR [95% CI] 0.69 [0.56,0.85]) and 38% (0.62 [0.48,0.80]) lower odds, respectively. Participants with a pre-pregnancy BMI > 30 kg/m^2^ had 1.81 times the odds of experiencing more severe COVID-19 compared to those with < 25 kg/m^2^ (1.81 [1.47,2.24]). Furthermore, more severe COVID-19 was observed for participants who worked in food service (2.08 [1.27,3.39]). Participants who worked in healthcare (0.74 [0.60,0.92]) or from home (0.80 [0.66,0.97]) were less likely to experience severe COVID-19.

Participants from lower economic status (poor, lower-middle class, middle class) had increased odds of more severe COVID-19 than wealthy participants, and odds increased with decreasing wealth (economic status: reference wealthy; poor: 2.72 [2.01,3.69]; lower-middle class: 2.07 [1.62,2.65]; middle class: 1.62 [1.29,2.04]). The same trend was seen with country-level economic status with participants from upper-middle class income countries having higher odds of more severe COVID-19 relative to those of high-income countries (reference high income; upper middle income: 1.77 [1.18, 2.65]).

A similar gradient was seen with education, where participants with high school (1.68 [1.39,2.03]) or less than high school education (1.77 [1.25,2.51]) had higher odds of more severe COVID-19 relative to participants with a graduate education (Fig. 3). Participants with more than one child (multiparous) and those with high-risk pregnancy had increased odds of more severe COVID-19 relative to participants with their first pregnancy and without a high-risk pregnancy. Finally, pre-pregnancy health status other than excellent also increased odds of more severe COVID-19, with highest odds among those with poorest health status.

#### Participants who enrolled after pregnancy

Only one-quarter of our analytic sample (*n* = 1063) were participants enrolled after pregnancy (the recently pregnant group). Generally, participant characteristics associated with greater COVID-19 severity were similar in participants in this category to those who were pregnant at enrollment. Having a high pre-pregnancy BMI (≥ 30 kg/m^2^), having poor economic status, a high-risk pregnancy, fair/poor pre-pregnancy health and having more than one previous pregnancy significantly increased the odds of more severe COVID-19 in this subgroup (Additional file 1: Appendix 3).

#### COVID-19 minimization behaviors and co-morbidities associated with COVID-19 severity

Among women with symptomatic COVID-19, self-reported behavioral characteristics around lowering risk for SARS-CoV-2 infection in the two weeks prior to infection were associated with increased COVID-19 severity for multiple measures in participants who enrolled during pregnancy. For example, participants who enrolled during pregnancy had an increased odds of more severe COVID-19 if they reported avoiding public gatherings and crowds in the two weeks prior to getting diagnosed with COVID-19 (Additional file 1: Appendix 4). During pregnancy, the presence of asthma was significantly associated with more severe COVID-19 relative to participants without asthma (2.15 [1.60,2.88]) (Additional file 1: Appendix 5). No increased odds of COVID-19 severity were seen in those with a history of high blood pressure or cardiovascular disease prior to pregnancy.

## Discussion

### Principal findings

In this study of 4,231 pregnant and recently pregnant participants with symptomatic COVID-19 across 41 countries, we demonstrated that social and demographic characteristics are associated with COVID-19 severity during pregnancy. Younger age, lower economic class (at individual and country level), lower educational attainment, and employment setting (food service vs. unemployed) were associated with increased odds of more severe COVID-19. In addition, we found several co-morbidities associated with COVID-19 severity: having a prior pregnancy vs. first pregnancy, pre-pregnancy higher BMI, asthma, and high-risk pregnancy all increase the odds of more severe COVID-19.

These results are similar to other studies in the non-pregnant population which have found a disproportionate burden of SARS-CoV-2 infection among persons living in poverty and those with lower educational attainment [21, 22]. In the pregnant population, various studies have also found a connection between socioeconomic disparities, and maternal morbidity and mortality of mothers infected with COVID-19 [3, 4, 23, 24]. Two studies noted that younger maternal age has been associated with an increased risk of COVID-19 infection, but did not find a relationship between age and COVID-19 severity among infected pregnant participants [6, 11]. Our study found that younger maternal age was associated with more severe COVID-19. Younger pregnant participants may delay seeking medical care assuming they are at a lower risk of severe COVID-19 or other diseases [2]. Another study of pregnant patients with COVID-19 infection who delivered in United States hospitals found that those with more severe illness had older mean age, higher median body mass index, and pre-existing medical comorbidities [25]. In that study, universal screening for infection was not routine across all clinical sites, and asymptomatic positive test results from screening were included in the COVID-19 severity outcome when available. Differences in findings around maternal age and COVID-19 severity could be a function of differences in sampling given IRCEP was an online global study compared to the single country clinical setting that included asymptomatic test results.

Similar to data from Sakowicz [11], our study found that prior pregnancy was also associated with increased risk of severity. We did not assess whether the age or number of living children at home is a risk factor for SARS-CoV-2 infection, or whether the number of children at home is a surrogate marker for other social factors such as crowdedness or increased exposures outside of the home. Children as well as other adults at home may partially explain the severity of infection among pregnant people.

While not the focus of our analysis, our results on comorbidities are congruent with data compiled in a meta-analysis reporting higher BMI (> 30), asthma, and any other pre-existing maternal comorbidity are factors associated with severe COVID-19 disease in pregnancy [26].

Epidemiological studies have reported a high risk of SARS-CoV-2 infection among food workers as a result of contact with one another for extended periods, including at work and shared housing and transportation [27]. The significant association we found in our study of working in the food industry with higher odds of COVID-19 severity is supported by a study by Mutambudzi et al. [28]. After adjusting for age, sex, ethnicity, and country of birth, people working in the food industry still had higher odds of COVID-19 severity [28]. Women make up a higher proportion than men in the food industry. For example, in the United States women comprise approximately 60% of food service workers [29]. We do not have further information on the specific type of employment; i.e., food processing, manufacturing, or agriculture, which limits our ability to contextualize the nuances of the risk. However, regardless of the type of setting, factors that contribute to high risk of infection could be further aggravated by the baseline social characteristics analyzed in this study resulting in a higher degree of COVID-19 severity. Another explanation for the higher odds of COVID-19 severity among people working in the food service industry is that food service workers in our study had lower socioeconomic factors and were younger than other groups. In our study, two-thirds of participants employed in food services reported being either poor or in the lower-middle class versus about one-third to half of participants reporting other occupations. Women employed in food services had a lower median age than other groups. When adjusting for employment status, economic class, and age in the model simultaneously, the adjusted odds ratios of these variables on the outcome of COVID-19 severity were attenuated but remained significant (not shown). No interactions were examined.

Individual lower income and lower educational attainment were associated with COVID-19 severity in Prasannan et al. [6]. In our study, we also found an association between lower country income (World Bank Classification) and COVID-19 severity in pregnant and recently pregnant participants. Those coexisting factors may play a large role in the association we observed. There is limited data from low/middle-income countries (LMICs) on the impact of SARS-COV-2 infection in pregnancy in our study. Pregnant people in LMICs are disproportionately affected by poor COVID-19 outcomes given co-morbidities and more limited healthcare access [4, 30].

Lastly, all the factors highlighted in this analysis (age, education, income, comorbidities) contribute in one way or another to perpetuating the cycle of disease in populations already at risk. COVID-19 has had a profound impact on our society and has exacerbated the maternal health crisis existing in many areas of the world. Pregnant individuals, already facing disadvantaged situations as a result of unfavorable sociodemographic conditions, are placed at higher risk of COVID-19 severity [31]. Recognizing the disparities in pregnant individuals with SARS-CoV-2 infection is imperative to provide an adequate and timely response that decreases additional burden at an individual, societal, and healthcare system level.

### Strengths and limitations

A strength of our analysis is its size and breadth, contributing important information on the relationships between social and behavioral characteristics with COVID-19 severity in a multinational registry with 4,231 participants in 41 countries. To our knowledge, IRCEP is the largest COVID-19 pregnancy registry. Although our sample is large, it may have limited generalizability to the general population of pregnant people, in that enrollment was conducted on line and require access to the Internet via computer/mobile phone.

Selection into the study population may be associated with the severity of COVID-19, and enrollment was allowed following birth or a pregnancy loss. We stratified by timing of enrollment (pregnant vs. recently pregnant) given differences in COVID-19 severity and disproportionate numbers of severe COVID-19 in the recently pregnant group. The recently pregnant group is composed of participants who enrolled retrospectively after pregnancy, including those who safely delivered their baby or experienced a pregnancy loss. The retrospective enrollment could have been related to SDHs and/or COVID-19 severity, which could explain any differences across analyses by enrollment timing. Also, those with severe COVID-19 late in pregnancy may not have had the ability to participate in the study until they recovered and, by then, they had delivered. In addition, given that the majority of the sample were those 25 to 34 years old (66%), selection bias may have affected the association between younger age and more severe COVID-19.

Our planned analysis focused on demographic and social characteristics. All of the data was self-reported, and the study had a potential for information bias, including COVID-19 severity and participant COVID-19 risk minimizing behaviors. Participant-reported behaviors to lower risk of SARS-CoV-2 infection seemed to increase the odds of more severe COVID-19, as the analysis conditions on having a symptomatic infection. This finding was thought to be related to collider bias [32], where the relationship between preventive behaviors and COVID-19 severity could have been distorted by conditioning on a positive test or symptoms. Our analysis examining the relationship between SDHs and COVID-19 severity was limited to positive, symptomatic COVID-19 diagnosis to minimize collider bias. As testing was not available to everyone, the inclusion of individuals with negative tests and asymptomatic COVID-19 tests would have been related to risk factors for COVID-19 infection and social factors of interest, including economics and occupation [15, 33]. Lastly, participants may have provided more socially acceptable responses versus admitting to not adhering to risk-minimization behaviors (Additional file 1: Appendix 4).

There were some limitations on the inclusion of SDHs. About 25% of patients eligible for this analysis were excluded due to missing social and demographic characteristics, as the demographic module was optional (Fig. 1). While self-reported socioeconomic status may be somewhat subjective, both lower self-reported wealth and lower World Bank country-level income were associated with increased severity of COVID-19 infection in this multinational study. The IRCEP registry did not capture detailed information on race or ethnicity on a global scale due to challenges in capturing this meaningfully across all the included countries. Race and ethnicity are correlated with other SDHs [34, 35], and the effect of race and ethnicity on COVID-19 severity may be explored in future work relating to specific countries.

In examining the relationship between social and demographic characteristics with COVID-19 severity at enrollment, we selected characteristics prior to pregnancy and prior to COVID-19 diagnosis where possible. However, temporality may not always be precisely known or available (e.g., employment questions during the pandemic in relation to COVID-19 infection and pregnancy). Finally, COVID-19 testing was not available in all regions, and hence SARS-CoV-2 infection status is subject to misclassification. Restricting to those with COVID-19 testing (94% of sample) did not appreciably modify the results. Despite the potential for misclassification, our findings are robust given the requirement for participants in this analysis to exhibit COVID-19 symptoms.

The social and demographic characteristics examined in this study are expected to be correlated with one another and with underlying risk factors for COVID-19 severity. Fitting an adjusted model combining social and demographic characteristics was limited to including occupation, economic class, and maternal age when exploring the increased risk of more severe infection among food service workers given that our primary interest was on individual characteristics. In addition, sample size limitations in the severe COVID-19 category (~ 100 participants) limited the number of characteristics that could be adjusted in multivariable modeling.

Despite the limitations, this study provides important insights into the relationship between demographic factors and SDHs at increased odds of more severe COVID-19 during pregnancy prior to introduction of COVID-19 vaccines.

## Conclusions

Demographic, social, and structural determinants of health that have been reported in the general population to be associated with an increased odds of COVID-19 severity were also associated with a higher odd of COVID-19 severity in pregnancy. Further exploration of structural factors related to socioeconomic inequity is required to improve the delivery of timely intervention and optimal healthcare for all pregnant populations.

## Supplementary Information

**Additional file 1.**

## Data Availability

The study data are available through Pregistry. Requests for sharing should be sent to the Corresponding Author and will be evaluated on an individual basis.

## Abbreviations

ARDS: Acute respiratory distress syndrome
BMI: Body mass index
CDC: Centers for Disease Control and Prevention
CI: Confidence interval
COVID-19: The coronavirus disease 2019
ECMO: Extracorporeal membrane oxygenation
ENCePP: European Network of Centres for Pharmacoepidemiology and Pharmacovigilance
ICU: Intensive care unit
IRCEP: International Registry of Coronavirus Exposure in Pregnancy
LMICs: Low/middle-income countries
MERS: Middle Eastern Respiratory Syndrome
OR: Odds ratio
SARS: Severe acute respiratory syndrome
SD: Standard deviation
SDHs: Social determinants of health
