# Supplementary material for: Social determinants of health associated with COVID-19 severity during pregnancy: a multinational cohort study (in the International Registry of Coronavirus Exposure in Pregnancy)
Source: BMC Public Health. 2022 Dec 3;22:2256. doi: 10.1186/s12889-022-14532-8 (PMC9719160; doi:10.1186/s12889-022-14532-8)
Supplement: Supplementary file 1 — Additional file 1. [file 12889_2022_14532_MOESM1_ESM.docx]

## Appendix 1 Classification of COVID-19 Severity

**Severity of COVID-19** was defined based on the definitions used in the IRCEP quarterly reports.  Among those who are COVID-19 positive (positive test or clinical confirmation), the severity will be determined as follows (4-levels):

**LEVEL 1 – Asymptomatic:** No symptoms (asymptomatic but COVID-19 positive per testing) Note: these individuals will not be included in any analyses based on specification of our analysis population.

**LEVEL 2 – Mild:** Symptoms but not meeting the definition of moderate or severe

**LEVEL 3 -Moderate**

Any **one** of:

- Abnormal chest-X-ray or CT scan
- Acute respiratory distress syndrome
- Difficulty breathing or shortness of breath
- Pneumonia
- Visited the Emergency Department (not hospitalized) **OR** Visited a clinic or hospital **AND** any **one** of:
- Persistent pain or pressure in the chest
- Bluish lips or face
- Visited the Emergency Department (not hospitalized) **OR** Visited a clinic or hospital **AND** any **two** of:
- Highest fever (≥38.0°C or ≥100.4°F)
- Sore throat OR Cough OR Runny nose
- Fatigue
- Headache
- Muscle aches (myalgia)
- Loss of smell OR Loss of taste
- Abdominal pain OR Diarrhea OR Nausea or vomiting

**LEVEL 4 - Severe**:

Any **one** of:

- Was admitted into the intensive care unit (ICU)
- Needed respiratory assistance/Mechanical ventilation/ECMO
- Was hospitalized **AND**experienced any one of:
- Organ failure
- Acute respiratory distress syndrome (ARDS)
- Bluish lips or face
- Difficulty breathing or shortness of breath
- Persistent pain or pressure in the chest
- Pneumonia

## Appendix 2: COVID-19 Symptoms and Hospitalization at Enrollment with Frequency <50%

|  | **Total**  **(n= 4,231)** | **Currently Pregnant**  **(n= 3,168)** | **Recently Pregnant**  **(n= 1,063)** |
| --- | --- | --- | --- |
|  |  | **n (%)** |  |
| **COVID-19 Symptoms** |  |  |  |
| Abdominal Pain | 608 (14.4) | 469 (14.8) | 139 (13.1) |
| Acute respiratory distress syndrome | 227 (5.4) | 139 (4.4) | 88 (8.3) |
| Bluish lips or face | 67 (1.6) | 54 (1.7) | 13 (1.2) |
| Difficulty breathing or shortness of breath | 1,628 (38.5) | 1,169 (36.9) | 459 (43.2) |
| Persistent pain or pressure in the chest | 703 (16.6) | 511 (16.1) | 192 (18.1) |
| Sudden confusion | 269 (6.4) | 183 (5.8) | 86 (8.1) |
| Abnormal chest-X-ray or CT scan | 217 (5.1) | 109 (3.4) | 108 (10.2) |
| Diarrhea | 1,257 (29.7) | 961 (30.3) | 296 (27.8) |
| Fever | 1,554 (36.7) | 1,085 (34.2) | 469 (44.1) |
| Diarrhea, Nausea or Vomiting, and/or Abdominal Pain | 2,012 (47.6) | 1,544 (48.7) | 468 (44.0) |
| Headache | 358 (8.5) | 296 (9.3) | 62 (5.8) |
| High Fever (>= 38˚C) | 1,047 (24.7) | 694 (21.9) | 353 (33.2) |
| Nausea or vomiting | 1,320 (31.2) | 1041 (32.9) | 279 (26.2) |
| Organ failure | 14 (0.3) | 5 (0.2) | 9 (0.8) |
| Other COVID-19 symptoms | 642 (15.2) | 499 (15.8) | 143 (13.5) |
| Pneumonia | 184 (4.3) | 83 (2.6) | 101 (9.5) |
| Sore Throat | 1,992 (47.1) | 1,475 (46.6) | 517 (48.6) |
| **COVID-19-related Hospitalization** |  |  |  |
| Needed respiratory assistance/Mechanical ventilation/ECMO | 84 (2.0) | 39 (1.2) | 45 (4.2) |
| Hospitalized | 310 (7.3) | 131 (4.1) | 179 (16.8) |
| Admitted to ICU | 66 (1.6) | 16 (0.5) | 50 (4.7) |

## Appendix 3. Recently Pregnant Participant Characteristics Associated with COVID-19 Severity


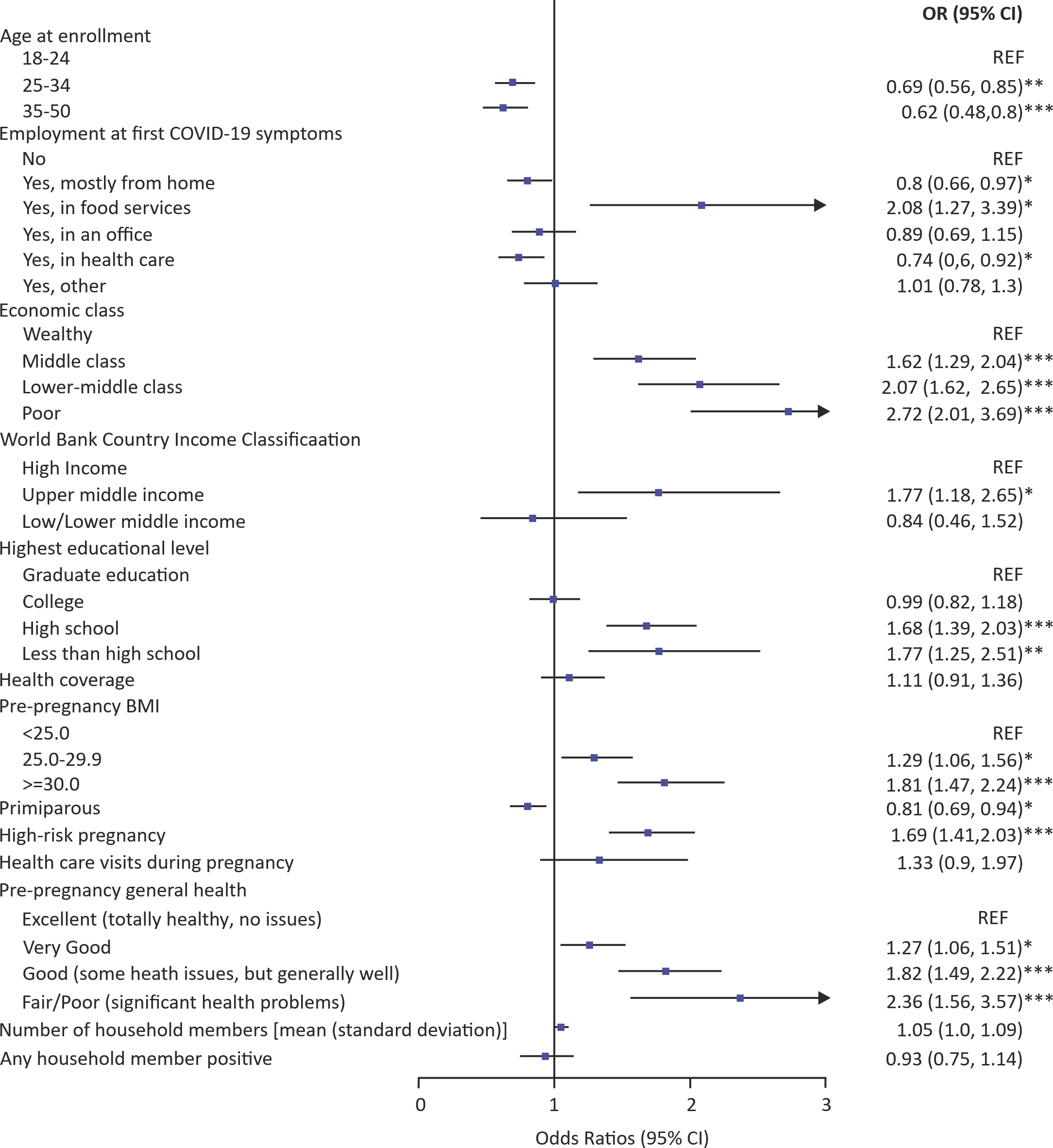


*p≤0.05, **p≤0.01. and ***p≤0.001

Note: Univariate mixed-effects ordinal logistic regression models with country as a random effect. Odds ratios (OR), Wald-type 95% confidence intervals, and Benjamini-Hochberg adjusted p-values were reported.

## Appendix 4. COVID-19 Preventive Behaviors

## Appendix 4A: COVID-19 Preventive Behaviors Prior to Enrollment

|  | **Total** | **Currently Pregnant** | **Recently Pregnant** |
| --- | --- | --- | --- |
|  |  | **n (%)** |  |
| **COVID-19 Preventative Behaviors** |  |  |  |
| Avoided public spaces, gatherings, or crowds | 2863 (67.7) | 2126 (67.1) | 737 (69.3) |
| Avoided contact with people who could be high-risk | 2583 (61.0) | 1939 (61.2) | 644 (60.6) |
| Avoided eating at restaurants | 2273 (53.7) | 1662 (52.5) | 611 (57.5) |
| Canceled a doctor's appointment | 545 (12.9) | 403 (12.7) | 142 (13.4) |
| Disinfected surfaces around me | 2498 (59.0) | 1845 (58.2) | 653 (61.4) |
| Canceled or postponed personal or social activities | 1582 (37.4) | 1126 (35.5) | 456 (42.9) |
| Washed my hands with soap or used hand sanitizer several times per day | 3765 (89.0) | 2847 (89.9) | 918 (86.4) |
| Wore a face mask | 3603 (85.2) | 2838 (89.6) | 765 (72.0) |
| Prayed | 1370 (32.4) | 992 (31.3) | 378 (35.6) |
| Stockpiled food or water | 695 (16.4) | 480 (15.2) | 215 (20.2) |
| Stockpiled medication | 218 (5.2) | 151 (4.8) | 67 (6.3) |
| Stockpiled hand sanitizer or disinfectant wipes | 817 (19.3) | 575 (18.2) | 242 (22.8) |
| Canceled or postponed air travel for pleasure | 573 (13.5) | 434 (13.7) | 139 (13.1) |
| Canceled or postponed air travel for work | 357 (8.4) | 257 (8.1) | 100 (9.4) |
| Visited a doctor | 1189 (28.1) | 858 (27.1) | 331 (31.1) |
| Worked or studied at home | 1408 (33.3) | 1049 (33.1) | 359 (33.8) |
| Canceled or postponed work or school activities | 947 (22.4) | 678 (21.4) | 269 (25.3) |

**Appendix Table 4B: Preventive Behaviors and Association with COVID-19 Severity at Enrollment (Pregnant at Enrollment Group)**

|  | **Mild** | **Moderate** | **Severe** |  |
| --- | --- | --- | --- | --- |
| **COVID-19 Preventative Behaviors** | **n (%)** | | | **OR (95% CI)** |
| Avoided public spaces, gatherings, or crowds | 826 (63.0) | 1208 (69.3) | 92 (80.0) | 1.4 (1.2,1.63)*** |
| Avoided contact with people who could be high-risk | 768 (58.6) | 1096 (62.9) | 75 (65.2) | 1.16 (1.0,1.34) |
| Avoided eating at restaurants | 650 (49.6) | 944 (54.2) | 68 (59.1) | 1.19 (1.03,1.37)* |
| Canceled a doctor's appointment | 163 (12.4) | 227 (13.0) | 13 (11.3) | 0.97 (0.79,1.21) |
| Disinfected surfaces around me | 734 (56.0) | 1039 (59.6) | 72 (62.6) | 1.16 (1.0,1.34) |
| Canceled or postponed personal or social activities | 424 (32.3) | 657 (37.7) | 45 (39.1) | 1.24 (1.07,1.44)* |
| Washed my hands with soap or used hand sanitizer several times per day | 1180 (90.0) | 1565 (89.8) | 102 (88.7) | 1.01 (0.8,1.28) |
| Wore a face mask | 1164 (88.8) | 1573 (90.3) | 101 (87.8) | 1.02 (0.81,1.29) |
| Prayed | 357 (27.2) | 594 (34.1) | 41 (35.7) | 1.19 (1.01,1.4) |
| Stockpiled food or water | 173 (13.2) | 286 (16.4) | 21 (18.3) | 1.35 (1.1,1.64)* |
| Stockpiled medication | 43 (3.3) | 98 (5.6) | 10 (8.7) | 1.97 (1.39,2.79)*** |
| Stockpiled hand sanitizer or disinfectant wipes | 222 (16.9) | 323 (18.5) | 30 (26.1) | 1.27 (1.05,1.53)* |
| Canceled or postponed air travel for pleasure | 166 (12.7) | 255 (14.6) | 13 (11.3) | 1.08 (0.88,1.33) |
| Canceled or postponed air travel for work | 84 (6.4) | 162 (9.3) | 11 (9.6) | 1.48 (1.14,1.93)* |
| Visited a doctor | 259 (19.8) | 559 (32.1) | 40 (34.8) | 1.68 (1.42,1.98)*** |
| Worked or studied at home | 440 (33.6) | 570 (32.7) | 39 (33.9) | 0.92 (0.79,1.07) |
| Canceled or postponed work or school activities | 254 (19.4) | 395 (22.7) | 29 (25.2) | 1.2 (1.01,1.43) |

Footnote: To characterize the association of each social and demographic characteristic with COVID-19 severity (Mild, moderate, severe), univariate mixed-effects ordinal logistic regression modeling was used. Each model accounted for country of residence as a random effect to estimate odds ratios (OR) and 95% confidence intervals (CI). Adjusted p-values using the Benjamini-Hochberg method were tabulated to adjust for multiple testing. *p≤0.05, **p≤0.01. and ***p≤0.001

**Appendix Table 4C: Preventive Behaviors and Association with COVID-19 Severity at Enrollment (Recently Pregnant at Enrollment Group)**

|  | **Mild** | **Moderate** | **Severe** |  |
| --- | --- | --- | --- | --- |
| **COVID-19 Preventative Behaviors** | **n (%)** | | | **OR (95% CI)** |
| Avoided public spaces, gatherings, or crowds | 277 (71.9) | 346 (66.5) | 114 (72.2) | 0.86 (0.67,1.11) |
| Avoided contact with people who could be high-risk | 233 (60.5) | 314 (60.4) | 97 (61.4) | 0.98 (0.77,1.25) |
| Avoided eating at restaurants | 215 (55.8) | 299 (57.5) | 97 (61.4) | 1.15 (0.91,1.45) |
| Canceled a doctor's appointment | 56 (14.5) | 64 (12.3) | 22 (13.9) | 0.94 (0.66,1.32) |
| Disinfected surfaces around me | 224 (58.2) | 333 (64.0) | 96 (60.8) | 1.16 (0.91,1.48) |
| Canceled or postponed personal or social activities | 150 (39.0) | 241 (46.3) | 65 (41.1) | 1.15 (0.91,1.46) |
| Washed my hands with soap or used hand sanitizer several times per day | 331 (86.0) | 457 (87.9) | 130 (82.3) | 0.88 (0.62,1.24) |
| Wore a face mask | 274 (71.2) | 374 (71.9) | 117 (74.1) | 0.95 (0.72,1.25) |
| Prayed | 116 (30.1) | 196 (37.7) | 66 (41.8) | 1.47 (1.14,1.89)* |
| Stockpiled food or water | 69 (17.9) | 109 (21.0) | 37 (23.4) | 1.32 (0.99,1.77) |
| Stockpiled medication | 19 (4.9) | 31 (6.0) | 17 (10.8) | 1.8 (1.11,2.93) |
| Stockpiled hand sanitizer or disinfectant wipes | 70 (18.2) | 127 (24.4) | 45 (28.5) | 1.48 (1.13,1.96)* |
| Canceled or postponed air travel for pleasure | 54 (14.0) | 64 (12.3) | 21 (13.3) | 0.89 (0.63,1.26) |
| Canceled or postponed air travel for work | 41 (10.6) | 42 (8.1) | 17 (10.8) | 0.87 (0.58,1.3) |
| Visited a doctor | 79 (20.5) | 186 (35.8) | 66 (41.8) | 2.0 (1.54,2.59)*** |
| Worked or studied at home | 141 (36.6) | 160 (30.8) | 58 (36.7) | 0.86 (0.67,1.11) |
| Canceled or postponed work or school activities | 94 (24.4) | 131 (25.2) | 44 (27.8) | 1.1 (0.84,1.43) |

Footnote: To characterize the association of each social and demographic characteristic with COVID-19 severity (Mild, moderate, severe), univariate mixed-effects ordinal logistic regression modeling was used. Each model accounted for country of residence as a random effect to estimate odds ratios (OR) and 95% confidence intervals (CI). Adjusted p-values using the Benjamini-Hochberg method were tabulated to adjust for multiple testing. *p≤0.05, **p≤0.01. and ***p≤0.001

**Appendix 5: Medical Comorbidity in Pregnant and Recently Pregnant Women**

|  | **Mild** | **Moderate** | | **Severe** |  |
| --- | --- | --- | --- | --- | --- |
|  |  | **n (%)** | |  | **OR (95% CI)** |
| **Pregnant** |  |  | |  |  |
| Asthma | 56 (4.3) | 153 (8.8) | | 11 (9.6) | 2.15 (1.60,2.88)*** |
| High blood pressure (chronic, pre-gestational) | 33 (2.5) | 61 (3.5) | | 4 (3.5) | 1.24 (0.82,1.88) |
| Any cardiovascular condition (heart failure, ventricular septal defects, arrythmias) | 24 (1.8) | 32 (1.8) | | 5 (4.3) | 1.25 (0.74,2.12) |
| **Recently Pregnant** |  |  | |  |  |
| Asthma | 21 (5.5) | 30 (5.8) | 10 (6.3) | | 1.14 (0.69,1.89) |
| High blood pressure (chronic, pre-gestational) | 14 (3.6) | 20 (3.8) | 8 (5.1) | | 1.22 (0.67,2.21) |
| Any cardiovascular condition (heart failure, ventricular septal defects, arrythmias) | 5 (1.3) | 10 (1.9) | 6 (3.8) | | 1.9 (0.81,4.46) |

Adjusted p-values using the Benjamini-Hochberg method were tabulated to adjust for multiple testing.

*p≤0.05, **p≤0.01. and ***p≤0.001
